# P-2142. Risk Factors and Outcomes of Bloodstream Infections After Intestinal Transplant: A Retrospective Cohort Study

**DOI:** 10.1093/ofid/ofaf695.2305

**Published:** 2026-01-11

**Authors:** Ana Khazan, Zachary Yetmar, Aneela Majeed, Masato Fujiki

**Affiliations:** Cleveland Clinic Foundation, Cleveland, OH; Cleveland Clinic, Cleveland, OH; Cleveland Clinic Foundation, Cleveland, OH; Cleveland Clinic Foundation, Cleveland, OH

## Abstract

**Background:**

Bloodstream infections (BSIs) are frequent and serious complications following intestinal transplantation.

**Figure:**
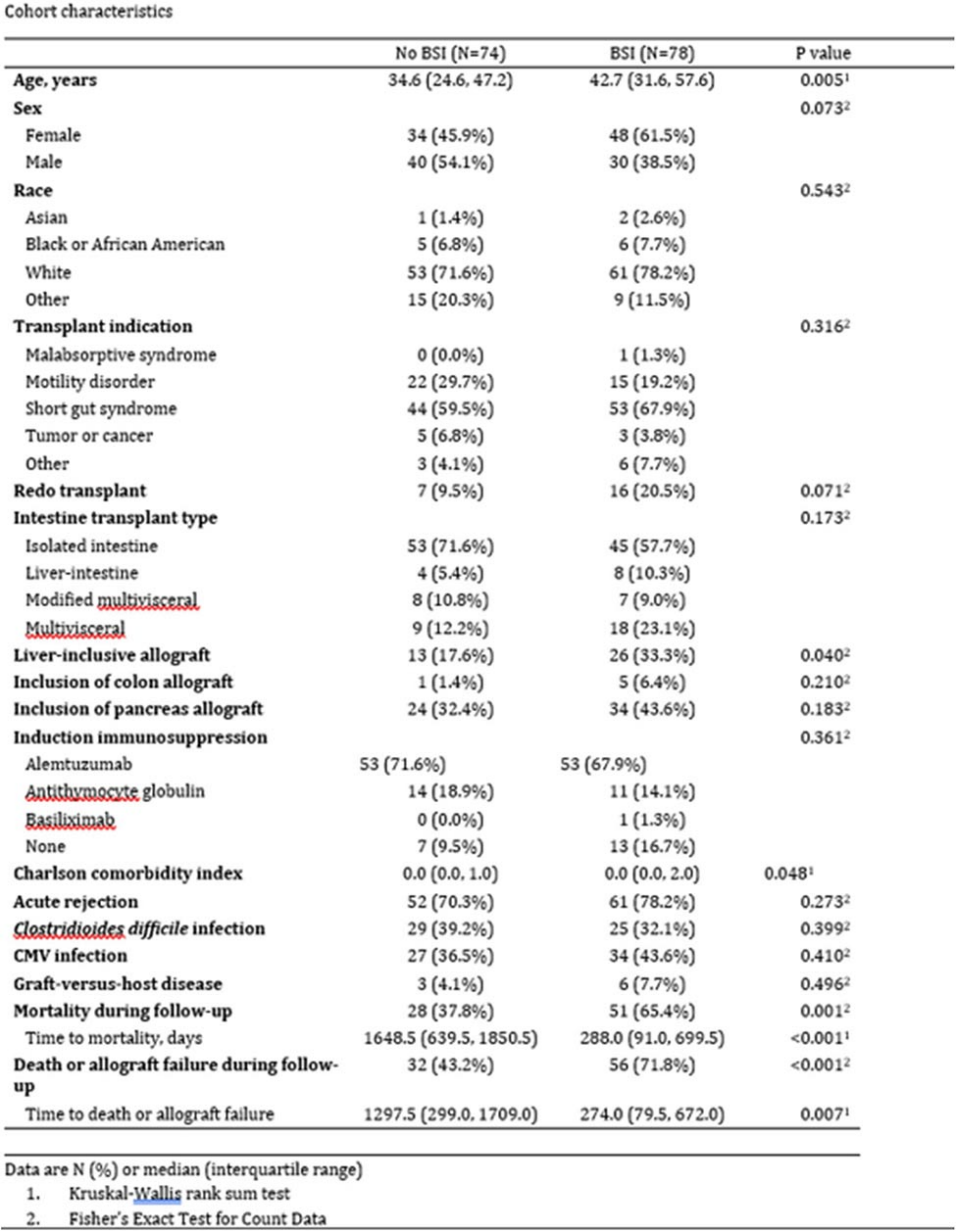
Descriptive Characteristics

**Figure:**
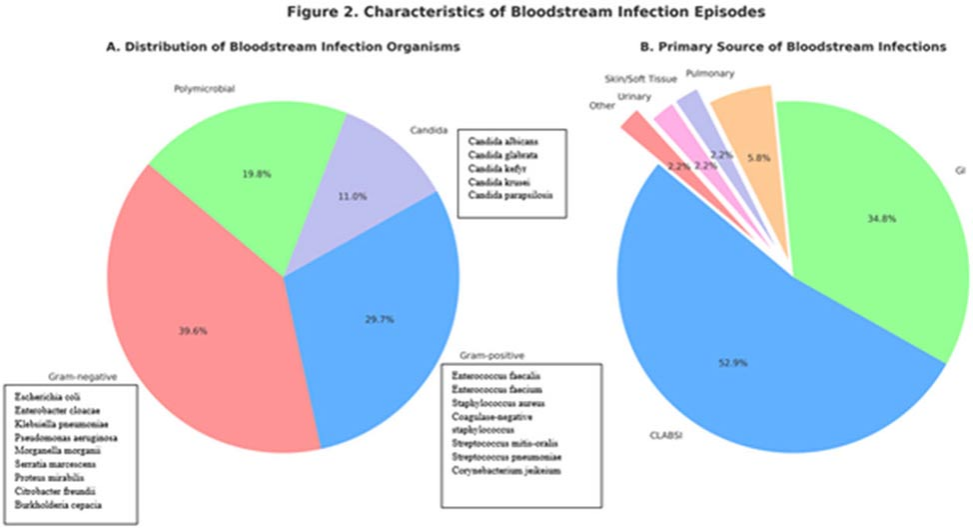
Characteristics of Bloodstream Infection Episodes Distribution and Primary Source of Bloodstream Infections

**Methods:**

We performed a retrospective study of 152 intestinal transplant episodes in 137 patients at a single center to characterize the incidence, risk factors, and outcomes of post-transplant BSI. The study period was from January 1, 2008 to December 31, 2022. Associations with BSI and death or graft failure were assessed by multivariable Cox regression.

**Figure:**
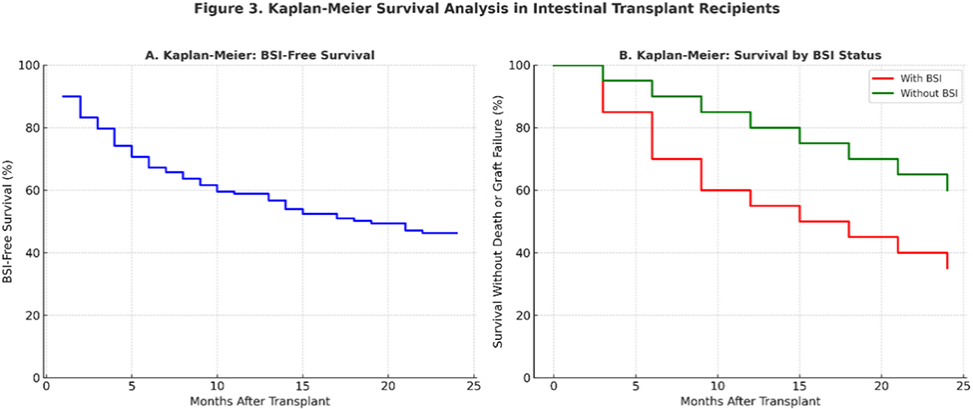
Kaplan-Meier Survival Analysis in Intestinal Transplant Recipients BSI-Free Survival and Survival by BSI Status

**Figure:**
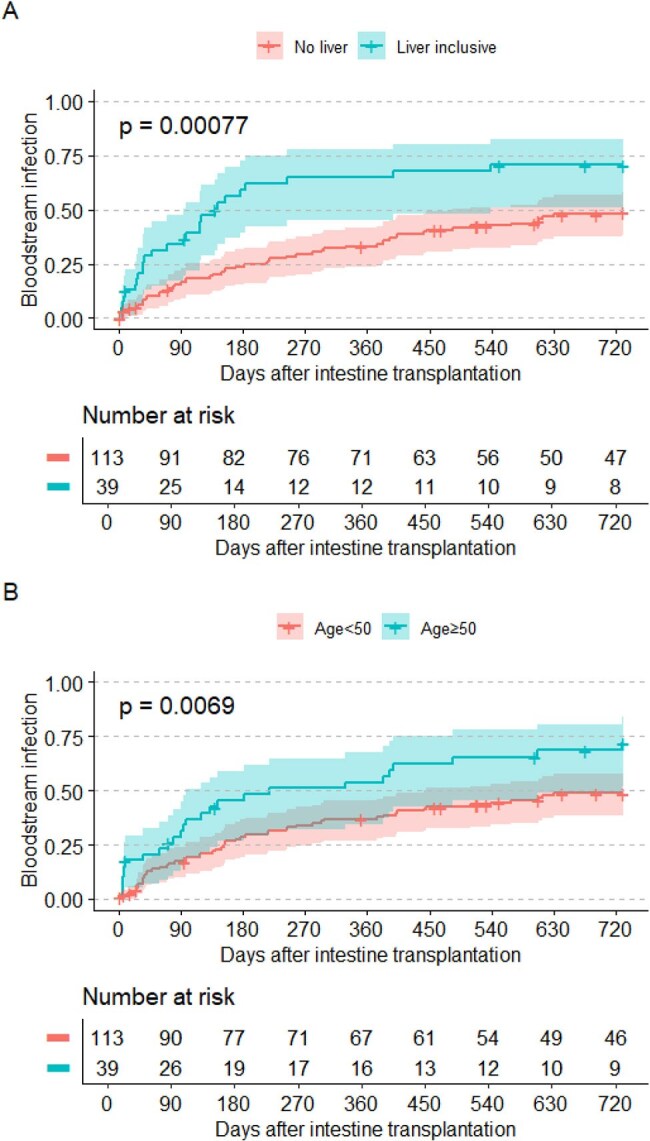
Kaplan-Meier Curve: Death or Graft Failure by BSI Status A. Death or Graft failure by Liver Inclusion B. Death or Graft failure by Age

**Results:**

78 transplant episodes experienced 140 BSIs. The cumulative incidence of BSI was 10.0% at 1 month, 32.8% at 6 months, and 53.7% at 24 months after transplant. Among 140 BSI episodes, 52.9% were catheter-associated, 34.8% gastrointestinal in origin and 12.3% from other sources. Polymicrobial infections occurred in 12.9% of episodes but accounted for 26.8% of all isolates. Of all isolates, 36.6% were gram-negative bacteria, 26.8% gram-positive, 9.9% *Candida* species, and 26.8% polymicrobial. Sixty percent developed sepsis, 54% required intensive care unit admission and 41% developed septic shock (defines as vasopressor requirement).

Significant risk factors for BSI included liver-inclusive allograft (HR 2.08, 95% CI 1.22–3.53, *p* = 0.007), acute rejection (HR 2.17, CI 1.24–3.81, *p* = 0.007), cytomegalovirus (CMV) infection (HR 1.94, CI 1.06–3.54, *p* = 0.030), increasing age (HR 1.02 per year, CI 1.01–1.04, *p* = 0.003), and female sex (HR 1.92, 95% CI 1.16-3.13, *p* = 0.011). Colon inclusion, C*. difficile* infection, alemtuzumab induction, and PTLD were not significantly associated with BSI. BSI was independently associated with death or graft failure (HR 5.32, 95% CI 3.34–8.46, *p*< 0.001).

**Conclusion:**

These findings highlight the need for targeted BSI prevention strategies, particularly in patients with liver-inclusive allografts, acute rejection, or CMV infection. Strategies aimed at reducing bloodstream infection risk may involve targeted catheter management and intensified monitoring during episodes of acute rejection and CMV infection. Given the strong association between BSI and adverse outcomes, future research should focus on optimizing infection prevention and management protocols to improve graft and patient survival in this vulnerable population.

**Disclosures:**

All Authors: No reported disclosures

